# The Relationship Between Penumbral Tissue and Blood-Brain Barrier Disruption in Acute Stroke Patients Presenting in an Extended Time Window

**DOI:** 10.3389/fneur.2020.582994

**Published:** 2020-12-08

**Authors:** Parisa Heidari, Sarah Blayney, Jarrhett Butler, Emi Hitomi, Marie Luby, Richard Leigh

**Affiliations:** National Institute of Neurological Disorders and Stroke, National Institutes of Health, Bethesda, MD, United States

**Keywords:** thrombolysis (tPA), extended time window, intracranial hemorrhage, blood-brain barrer, penumbra

## Abstract

**Background:** Penumbral brain tissue identified with multimodal imaging can be salvaged with reperfusion in an extended time window. The risk of severe hemorrhagic complications after reperfusion therapy increases with worsening disruption of the blood-brain barrier (BBB). The relationship between penumbral tissue and BBB disruption has not been previously studied.

**Methods:** Stroke patients presenting in an extended time window without a large vessel occlusion who underwent diffusion-perfusion MRI within 24 h of last-seen-normal were included. The volume of penumbral tissue was calculated using mismatch on MRI. Mean permeability derangement (MPD) of the BBB was measured within the ischemic lesion. A target profile (TP) for treatment was defined based on the EXTEND trial.

**Results:** 222 patients were included with a median age of 73 and 55% women. The median NIHSS was 6, the mean core volume was 14 ml, the mean ischemic volume was 47 mL and the mean mismatch volume was 33 mL. Higher MPD was significantly associated with less mismatch volume (*p* = 0.001). A target profile was associated with lower MPD (OR 0.97; CI 0.96:0.99; *p* < 0.001). Of the 105 patients who had a TP, 31 (30%) had a MPD > 20% suggesting an increased risk of hemorrhage. Thus, 33% (74/222) of patients had a favorable profile for benefit and safety.

**Conclusions:** Patients presenting in an extended time window with a favorable penumbral profile for treatment have less severe BBB disruption. Up to a third of patients who currently go untreated could be considered for enrollment in a clinical trial of thrombolysis in an extended time window.

## Background

The goal of a clinical trial is to determine if an intervention is safe and effective at its pre-specified objective. In acute stroke trials of reperfusion therapy, safety is primarily determined by the risk of symptomatic hemorrhagic complications, while efficacy is primarily determined by the ability to avert disability. It is well established that reperfusion of hypoperfused tissue that has not infarcted is an effective treatment for acute stroke (1). It has also been demonstrated that risk of hemorrhagic complications associated with reperfusion therapies increases with more severe disruption of the blood-brain barrier (BBB) (2, 3).

Currently there is no recommended reperfusion therapy for patients who present >4.5 h from symptom discovery in the absence of a large vessel occlusion. Recent trials using penumbral imaging have found that some of these patients may benefit from intravenous thrombolysis (4–6). However, it is not known if patients with a favorable pattern on penumbral imaging (favoring benefit) also have preserved integrity of their BBB (favoring safety). The purpose of this study was to investigate the relationship between penumbral profile and BBB disruption in patients presenting outside of the approved thrombolysis window who are ineligible for endovascular treatment.

## Methods

This research was conducted as a retrospective analysis of de-identified registry data, for which we obtained a determination of *Not Human Subjects Research* from the NIH Office of Human Subjects Research Protections (OHSRP).

### Population

The details of this population have been described in a previous publication (7). Briefly, it includes patients presenting to two stroke centers over a 5 year period who were evaluated and underwent MRI with diffusion weighted imaging (DWI) and perfusion weighted imaging (PWI) within 24 h of being last seen normal. Patients were excluded if they presented within 4 h of being last seen normal or if they received any acute reperfusion therapy. Four hours was used instead of 4.5 h under the assumption that patients presenting with a narrow window for treatment might be excluded due to time constraints. Patients with a large vessel occlusion (LVO) were excluded since there are established treatments for LVO in the extended time window. Patients were excluded if they did not have an ischemic lesion which was defined on PWI using a time-to-peak (TTP) threshold of 4 s beyond normal.

### Imaging

Details of the MRI protocol have been published previously (7). Ischemic lesions were identified on PWI using a TTP threshold of 4 s delay relative to the contralateral hemisphere. Relative delay in TTP has been found to be equivalent to other methods of identifying ischemia but does not require deconvolution of an arterial input function (AIF) making it less susceptible to errors introduced by AIF selection (8, 9). PWI lesions were superimposed on the apparent diffusion coefficient (ADC) maps after co-registration of the source images. The core infarct volume was defined as the portion of the ischemic lesion defined on PWI that had an ADC value <620 μm/s on DWI (1). The mismatch ratio was defined as the ischemic volume from PWI divided by the infarct core volume from DWI. The mismatch volume was defined as the PWI volume minus the DWI volume.

The mean permeability derangement (MPD), which is a measure of BBB disruption, was calculated from the source images of the PWI scan in the same manner previously described (7). PWI is generated using a dynamic susceptibility contrast (DSC) image sequence. In the setting of BBB disruption, the recorded signal in these images represents both intravascular flow and intraparenchymal leakage of gadolinium through the BBB. These two signals can be separated using an arrival time correction (10). The resulting metric, K_2_, is an index that reflects the fraction of the recorded signal that is due to gadolinium leakage through the BBB and can also be represented as a percent. Mean permeability derangement (MPD) is the average K_2_ value of all voxels within the ischemic lesion that are 2 standard deviations above normal identified from the contralateral hemisphere. It has previously been found that an MPD > 20% is associated with severe hemorrhagic complications after treatment with IV thrombolysis (2).

Target profile (TP) was defined using the parameters from the EXTEND trial (5). To be considered to have a TP, the mismatch ratio had to be >1.2, the mismatch volume had to be >10 mL and the core infarct had to be <70 mL. Image analysis was performed in Matlab (Mathworks, Natick, MA).

### Statistical Analysis

Mismatch volume was treated as an independent continuous variable and compared with MPD as a dependent continuous variable with linear regression. Mismatch ratio was not used in the linear regression due to its instability in certain situations (such as when it is infinite). TP was treated as a dichotomous variable and compared with MPD with logistic regression. Statistical analysis was performed in STATA 13 (StataCorp LLC, College Station, TX).

## Results

The cohort consisted of 222 patients with a median age of 73 of whom 55% were women. The median NIHSS was 6. The mean DWI core volume was 14 ml, the mean PWI ischemic volume was 47 mL and the mean mismatch penumbra volume was 33 mL. One hundred five patients (47%) met requirements for a TP. The median MPD was 18%.

**Figure 1:**
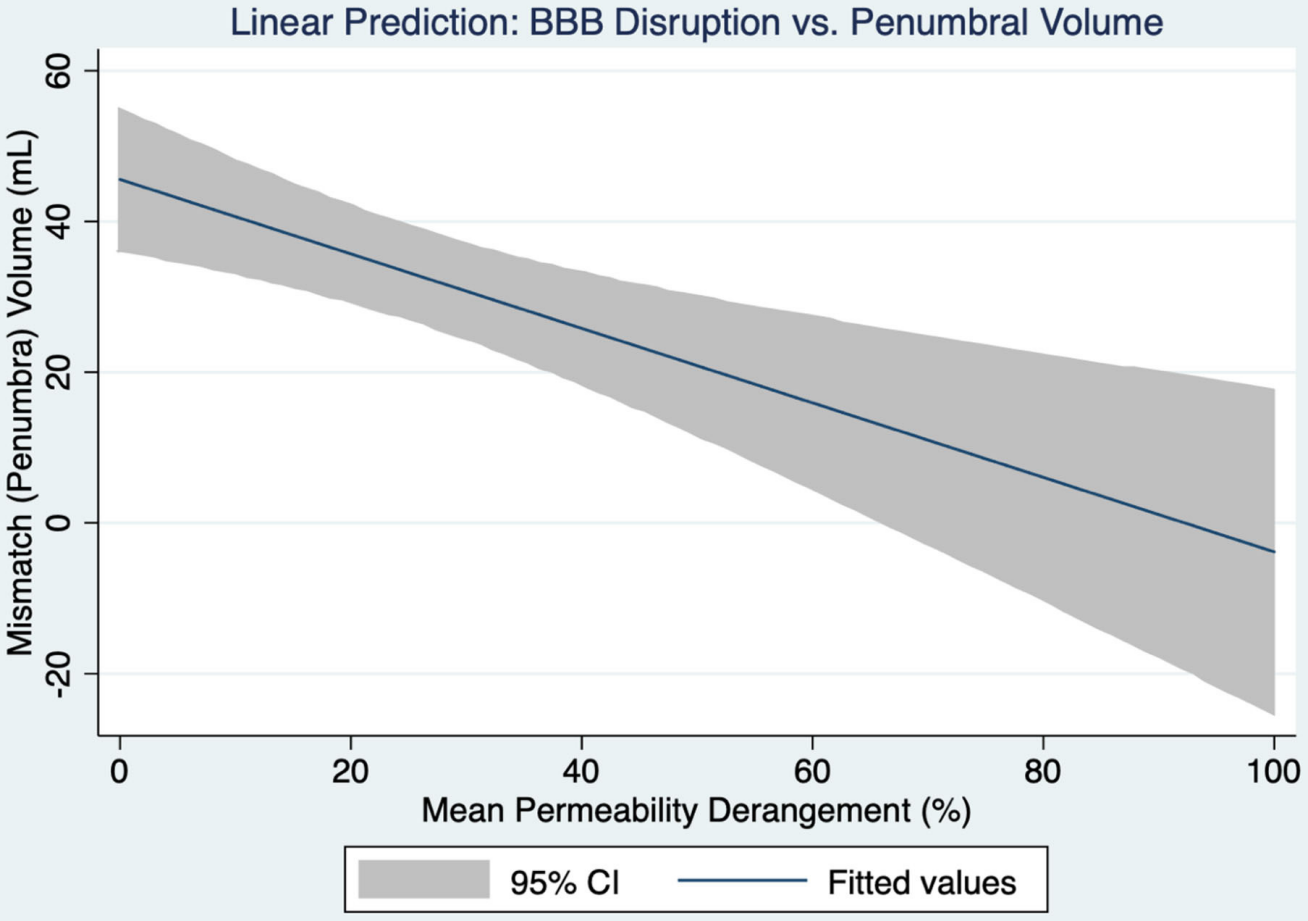
A linear prediction model for the relationship between blood-brain barrier disruption and the volume of penumbral tissue is shown with 95% confidence intervals.

Higher MPD was significantly associated with less mismatch volume (*p* = 0.001). Figure 1 shows the 95% confidence intervals for this relationship. In multivariate analysis mismatch volume remained significantly associated with MPD (*p* = 0.001) independent of age (*p* = 0.104), sex (*p* = 0.735), NIHSS (*p* = 0.868), DWI volume (*p* = 0.647) and time from symptom discovery to MRI (*p* = 0.724). Mismatch volume and PWI volume correlated highly and were not independent of each other.

**Figure 2:**
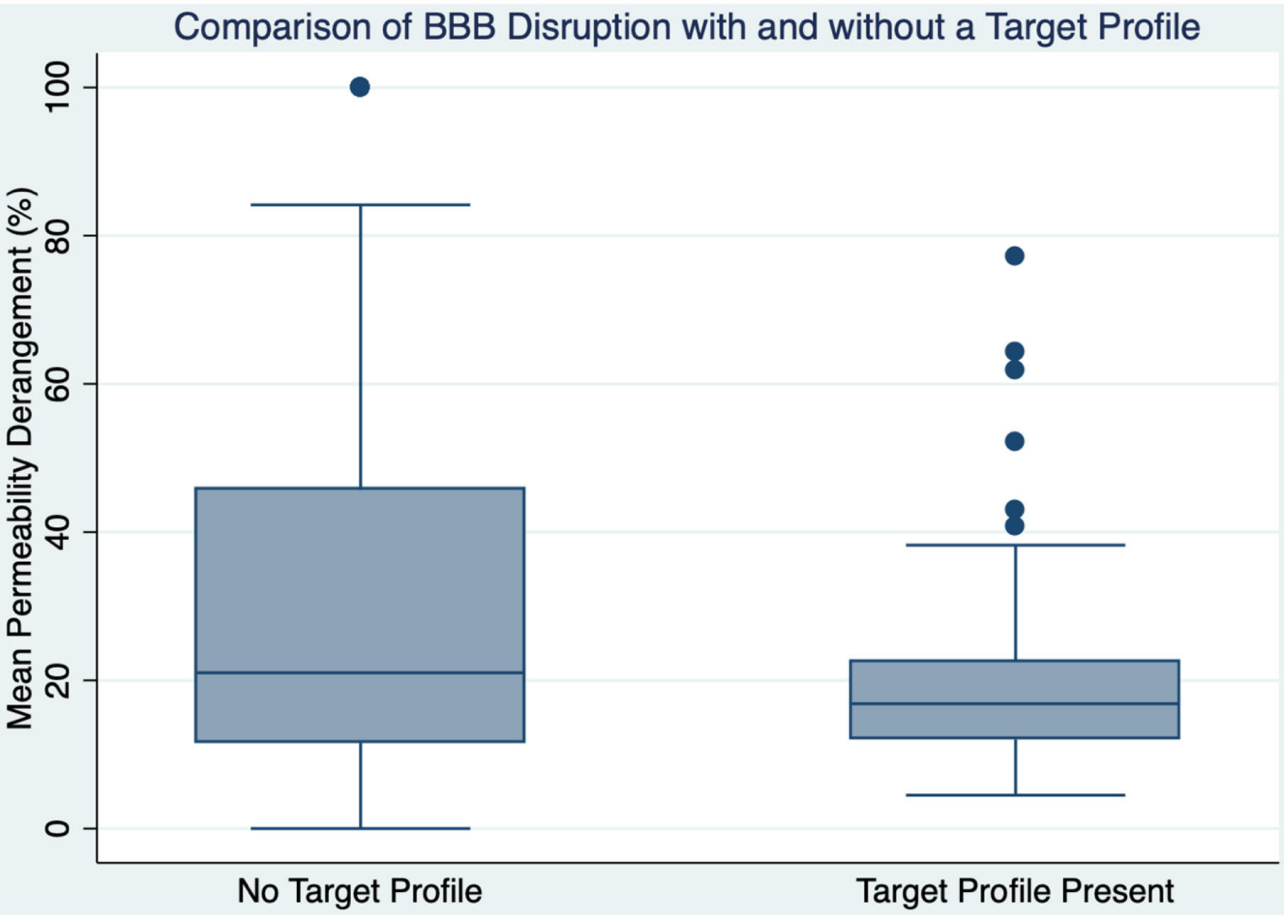
A boxplot is shown comparing blood-brain barrier disruption between those with and without a target profile.

TP was associated with lower MPD (OR 0.97; CI 0.96:0.99; *p* < 0.001) such that for every 10% increase in MPD the chance of having a TP is reduced by 30%. Figure 2 shows a boxplot of MPD for patients with and without a TP. Of the 105 patients who had a TP, 31 (30%) had a MPD > 20%. The 20% threshold has been associated with increased risk of parenchymal hematoma formation in patients treated with IV thrombolysis (2). If those patients were excluded, along with the patients who did not have a TP, the remaining 74 patients would potentially represent the population that would have maximum benefit while minimizing risk in a trial of extended time window thrombolysis. Taken over the 5-year period that this study was derived from, it implies that ~1.2 patients per month would be eligible for enrollment in such a study at our institutions.

To further evaluate the role of time in TP and MPD, the cohort was divided into two groups based on whether their time from symptom onset to MRI was greater than or less than 9 h. Two hundred eighteen patients had a documented time of symptom discovery; of these 176 were imaged <9 h from symptom discovery, and 42 were imaged >9 h from symptom discovery. Comparing these groups with logistic regression found that patients presenting in a later time window had significantly lower NIHSS (*p* = 0.001), but no difference in DWI volume, PWI volume, penumbra volume or MPD (Table 1). However, when treating time-to-MRI as a continuous variable and dichotomizing by the presence of a TP, later presentation was associated with decreased likelihood of a TP (OR 0.99, CI 0.997:0.999, *p* = 0.12). Comparing time-to-MRI with MPD > 20 did not find an association (*p* = 0.138) which is in agreement with our previously published findings (7).

**Table 1:** Population characteristics for all patients and the subgroups of patients imaged less than and greater than 9 h from the time of symptom discovery.

	**All patients (*n* = 222)**	**<9 h (*n* = 176)**	**>9 hours (*n* = 42)**	***p*-value**
Median age	73	75	66.5	0.05
Sex (% female)	55	57	50	0.43
Median NIHSS	6	9	3	0.001
Mean DWI volume (mL)	14	15	11	0.5
Mean PWI volume (mL)	47	48	43	0.69
Mean penumbra volume (mL)	33	33	32	0.9
Median MPD (%)	18	18	18	0.87
Mean time from symptom discovery to MRI (minutes)	364	242	875	–

## Discussion

In a broad sense this study asked the question: In a population of patients who presented in an extended time window without acute treatment options, was the presence of an imaging target for treatment benefit associated with an imaging target for treatment safety? In a narrower sense this study asked the question: Is the presence of penumbra associated with preserved BBB integrity? We found that most patients with an imaging target for benefit also had an imaging target for safety. Furthermore, a larger amount of penumbral tissue was associated with less disruption of the BBB.

The ischemic penumbra was originally defined as loss of electrical activity in brain tissue in the setting of decreased cerebral blood flow below a threshold such that this activity could be restored in the setting of restoration of blood flow (11). This concept of penumbra was later modified to reflect tissue at risk of infarction in the absence of reperfusion, and thus a target for salvage with acute reperfusion therapies. The introduction of MRI led to the development of a biomarker for penumbral tissue, the diffusion-perfusion mismatch (12). Advances in technology, combined with real-time post-processing services, made penumbral imaging more widely available using CT perfusion. Through a series of studies that culminated with the DEFUSE 3 study (1), penumbral imaging was validated not only as a way to select patients who would benefit from treatment, but also as a way to remove the restrictive time-based model for making treatment decisions, at least for mechanical thrombectomy (13).

Treatment with intravenous thrombolytics, however, remains time-based. Delay in arrival to the emergency room is the most common reason patients are not treated with thrombolysis (14). Recent advances using FLAIR MRI have expanded treatment of patients whose onset is unknown, such as wake-up stroke (15). However, using this approach, half of patients are still excluded due to being in an extended time window (16). Thus, recent studies have focused on using penumbral imaging to identify patients who would benefit from thrombolysis independent of time (5, 6). The ECASS 4 study was stopped early but found a trend to benefit when selecting patients based on a DWI/PWI mismatch. The EXTEND trial which was largely a CT-based trial found patients with penumbral tissue were more likely to have reduced disability when treated with thrombolysis. A meta-analysis of these studies strengthened the findings and reported a symptomatic hemorrhage rate of 5% (4).

**Figure 3:**
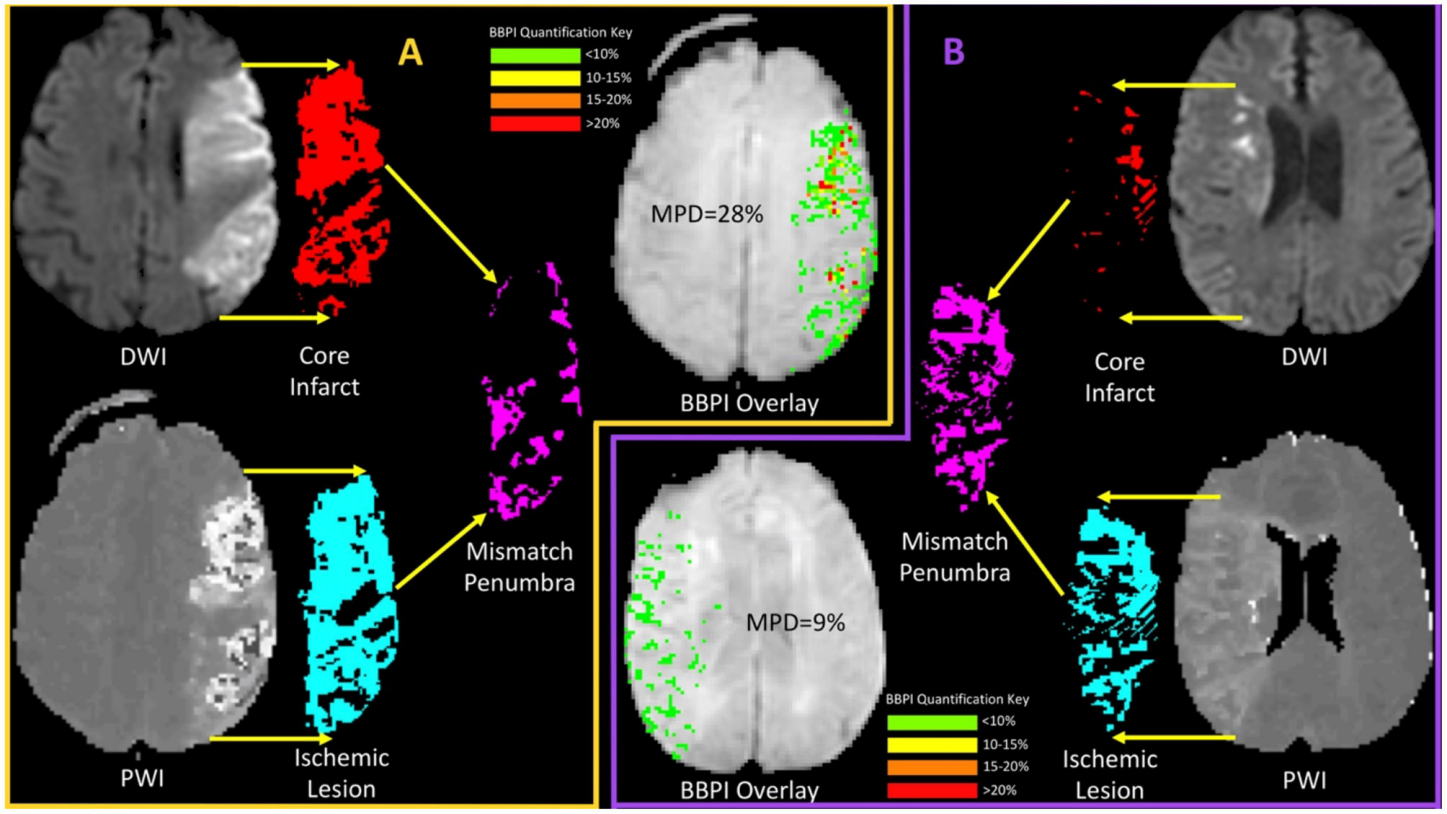
A comparison of mean permeability derangement (MPD) for two patients, one with and one without a target profile, is shown. **(A)** (orange boarder) shows an example of a patient with a large ischemic core (red) which when compared with the ischemic lesions (light blue) results in a small mismatch (magenta) relative to the core and a non-target profile. The BBPI overlay for this patient shows multiple focal areas of red that indicate >20% leakage resulting in an MPD of 28%. **(B)** (purple boarder) shows an example of a patient with a small core (red) and a much larger ischemic lesion (light blue) resulting in a large mismatch (magenta) and the presence of a target profile. For this patient the BBPI overlay is entirely green (<10%) resulting in an MPD of 9%. These examples demonstrate the finding of less BBB disruption in patients with a better penumbral profile.

Thus, despite treatment of patients out to 9 h, the hemorrhage rate remained modest. This could in part be due to the inclusion of wake-up strokes that were not actually in an extended time window. Another hypothesis is that patients with a favorable penumbral pattern at an extended time point have a similar hemorrhage risk compared to patients presenting in an early window. Our results support the latter. Specifically, patients with larger amounts of preserved ischemic tissue had less injury not only to that tissue, but the core infarct as well, when assessed by damage to the BBB. Figure 3 shows an example of this by comparing the BBB profiles of two patients, one with a large core that is not a TP, and one with a small core and large mismatch that is a TP. The preservation of penumbral tissue into extended time windows is thought to be facilitated by collateral blood flow, the robustness of which appears to vary widely throughout the population. It appears that these collaterals not only delay the growth of the infarct but may also prevent rupture of the BBB.

We also found that one third of patents presenting in an extended time window with a favorable penumbral pattern were potentially at high risk for severe hemorrhagic complications based on BBB disruption. This could be in part because we extended the window out to 24 h; however, based on the DEFUSE 3 trial, we know that it is imaging and not time that should be guiding decisions (13). It also may be the case that not all severe hemorrhagic events are symptomatic, since MPD does not account for this, thus the actual number of patients with symptomatic intracranial hemorrhage might be lower than 30%. However, our results suggest that combining BBB imaging with penumbral imaging may be a way to identify a subset of patients who are most likely to benefit from thrombolysis in an extended time window.

There are several limitations to this study. It is a retrospective study of a deidentified dataset with minimal clinical information about the subjects. While prior studies suggest that BBB measurements are a good surrogate for hemorrhage risk, this has never been prospectively tested. Furthermore, the MPD threshold of 20% is an approximation and the true threshold may be different. This study also only focused on BBB disruption within the ischemic tissue and did not take into consideration BBB disruption that may occur in reperfused tissue. The results of this study only apply to MRI selected patients as the K_2_ metric can only be extracted from MRI and not from CTP.

## Conclusions

Patients presenting in an extended time window with a favorable penumbral profile for treatment have less severe BBB disruption. This may explain why hemorrhage rates in extended window trials have been modest. The addition of BBB imaging to existing post-processing methods that calculate penumbra has the potential to improve safety. Future trials of extended time window thrombolysis are needed.

## Data Availability Statement

The raw data supporting the conclusions of this article will be made available by the authors, without undue reservation upon reasonable request and after appropriate approval.

## Ethics Statement

The studies involving human participants were reviewed and determined by NIH Office of Human Subjects Research Protections (OHSRP) to qualify as Not Human Subjects Research. Written informed consent from the patients/participants was not required to participate in this study in accordance with the national legislation and the institutional requirements.

## Author Contributions

PH, SB, JB, and EH: processed the data and revised the manuscript for intellectual content. ML: major role in data acquisition and revised the manuscript for intellectual content. RL: design and conceptualized study, analyzed the data and finalized the manuscript. All authors contributed to the article and approved the submitted version.

## Conflict of Interest

The authors declare that the research was conducted in the absence of any commercial or financial relationships that could be construed as a potential conflict of interest.
